# Quality of life of chronic kidney patients on hemodialysis and related factors*

**DOI:** 10.1590/1518-8345.3641.3327

**Published:** 2020-07-15

**Authors:** Carolina Renz Pretto, Eliane Roseli Winkelmann, Leila Mariza Hildebrandt, Dulce Aparecida Barbosa, Christiane de Fátima Colet, Eniva Miladi Fernandes Stumm

**Affiliations:** 1 Universidade Regional do Noroeste do Estado do Rio Grande do Sul, Departamento de Ciências da Vida, Ijui, RS, Brazil.; 2 Universidade Federal de Santa Maria, Campus CESNORS, Palmeira das Missões, RS, Brazil.; 3 Universidade Federal de São Paulo, Departamento de Enfermagem Clínica e Cirúrgica, São Paulo, SP, Brazil.

**Keywords:** Quality of Life, Depression, Nursing, Signs and Symptoms, Renal Insufficiency, Chronic, Medication Adherence, Qualidade de Vida, Depressão, Enfermagem, Sinais e Sintomas, Insuficiência Renal Crônica, Adesão à Medicação, Calidad de Vida, Depresión, Enfermería, Signos y Síntomas, Insuficiencia Renal Crónica, Cumplimiento de la Medicación

## Abstract

**Objective:**

to verify the association between the health-related quality of life of chronic renal patients on hemodialysis with sociodemographic, clinical, depression and medication adherence characteristics.

**Method:**

a cross-sectional study with 183 chronic renal patients undergoing hemodialysis in the state of Rio Grande do Sul, Brazil. A sociodemographic and clinical questionnaire, Kidney Disease and Quality of Life Short-Form, Beck Depression Inventory and Morisky Medication Adherence Scale - eight items were used. Among the variables, comorbidities, complications of kidney disease and intercurrences during and after hemodialysis were evaluated. The analysis was performed with descriptive and analytical statistics.

**Results:**

55.2% of the patients were 60 years old or older, 35.0% were hypertensive, with regular quality of life, average of 62.61. Scores below average in the dimensions of quality of life were mainly associated with repetitive infections and edema as complications of the disease, pain during hemodialysis and weakness afterwards. Low drug adherence resulted in a worse quality of life, impacting ten of the 20 dimensions evaluated and depression in all, except for patient satisfaction.

**Conclusion:**

reduced quality of life in this population is associated with depressive symptoms, complications such as repetitive infections, pain and anemia, weakness after the dialysis session and low medication adherence. Actions aimed at changing these factors can promote well-being.

## Abstract (pt)

**Objetivo:**

verificar a associação entre a qualidade de vida relacionada à saúde de pacientes renais crônicos em hemodiálise com as características sociodemográficas, clínicas, depressão e adesão medicamentosa.

**Método:**

pesquisa transversal com 183 pacientes renais crônicos em hemodiálise do Estado do Rio Grande do Sul, Brasil. Foram utilizados questionário sociodemográfico e clínico, *Kidney Disease and Quality of Life Short-Form*, Inventário de Depressão de Beck e Escala de Adesão à Medicação de Morisky - oito itens. Entre as variáveis foram avaliadas comorbidades, complicações da doença renal e intercorrências durante e após a hemodiálise. A análise foi feita com estatística descritiva e analítica.

**Resultados:**

55,2% dos pacientes tinham 60 anos ou mais, 35,0% eram hipertensos, com qualidade de vida regular, média de 62,61. Escores abaixo da média nas dimensões de qualidade de vida foram associados, principalmente, às infecções repetitivas e ao edema como complicações da doença, dor durante a hemodiálise e fraqueza após. A baixa adesão medicamentosa repercutiu em uma pior qualidade de vida com impacto em dez das 20 dimensões avaliadas e depressão em todas, exceto satisfação do paciente.

**Conclusão:**

a qualidade de vida reduzida nessa população associa-se aos sintomas depressivos, complicações como infecções repetitivas, dor e anemia, fraqueza após a sessão dialítica e baixa adesão medicamentosa. Ações direcionadas à modificação desses fatores podem promover bem-estar.

## Abstract (es)

**Objetivo:**

verificar la asociación entre la calidad de vida relacionada con la salud de los pacientes renales crónicos sometidos a hemodiálisis con las características sociodemográficas, clínicas, de depresión y de adhesión a la medicación.

**Método:**

estudio transversal con 183 pacientes renales crónicos sometidos a hemodiálisis en el estado de Rio Grande do Sul, Brasil. Se utilizaron cuestionario sociodemográfico y clínico, *Kidney Disease and Quality of Life Short-Form*, Inventario de Depresión de Beck y Escala de adhesión a la Medicación Morisky - ocho ítems. Entre las variables, se evaluaron las comorbilidades, las complicaciones de la enfermedad renal y las complicaciones durante y después de la hemodiálisis. El análisis se realizó con estadística descriptiva y analítica.

**Resultados:**

el 55.2% de los pacientes tenían 60 años o más, 35,0% eran hipertensos, con calidad de vida regular, promedio de 62.61. Puntuaciones por debajo del promedio en las dimensiones de calidad de vida se asociaron, principalmente, con infecciones repetitivas y edema como complicaciones de la enfermedad, dolor durante la hemodiálisis y debilidad posterior. La baja adhesión a los medicamentos repercutió en una peor calidad de vida, afectando diez de las 20 dimensiones evaluadas y la depresión en todos, excepto la satisfacción del paciente.

**Conclusión:**

la calidad de vida reducida en esta población se asocia con síntomas depresivos, complicaciones como infecciones repetitivas, dolor y anemia, debilidad después de la sesión de diálisis y baja adhesión a la medicación. Las acciones dirigidas a cambiar estos factores pueden promover el bienestar.

## Introduction

The number of deaths caused by Chronic Kidney Disease (CKD) is increasing worldwide and, in 2017, 1,230.2 million people died^(^ 1 ^)^. In Brazil, in the same year, the number of deaths of patients on dialysis was estimated at 25,187 deaths, which represented a gross mortality rate of 19.9%. In the country, to ensure survival and treatment of patients, the number of active dialysis centers has increased and, among the modalities of renal replacement therapies offered, hemodialysis is predominant. In 2017, 93.1% of the patients were on this therapy^(^ 2 ^)^.

CKD is characterized by decreased renal function, explained by a glomerular filtration rate of less than 60 ml/min/1.73 m^2^ and/or markers of renal damage lasting three months or more^(^ 3 ^)^. Progressively, it becomes a metabolic and endocrine problem that triggers inflammation and compromises immune capacity. Patients affected by this disease have low socioeconomic conditions, a high risk of morbidity, mortality and lower Health-Related Quality of Life - HRQoL^(^ 3 ^-^ 5 ^)^.

HRQoL is the subject’s perception in relation to his position in life, cultural environment and values in which he is inserted, objectives, expectations, standards and concerns. It is related to physical health, mental state, independence, social relationships, beliefs and peculiarities of the environment^(^ 6 ^)^. So, it comprises effects of the disease and/or treatment in the diverse life dimensions.

Psychosocial and biological changes related to dialysis treatment increase the risk of developing depression in patients with CKD. It is estimated that this population has rates of this disorder being three to four times higher than the general population and two to three times higher than in individuals with other chronic diseases. Depression also increases the risk of progression of kidney disease, worse clinical outcomes and mortality^(^ 7 ^)^.

Although the relationship between depression and HRQoL in CKD is well established^(^ 8 ^-^ 9 ^)^, it can be deepened with respect to the commitment of each domain that integrates it. Studies with chronic renal patients undergoing hemodialysis have also shown an association between quality of life, sociodemographic characteristics^(^ 10 ^-^ 12 ^)^ and comorbidities^(^ 13 ^)^. Regarding the relationship with complications of kidney disease, few studies have attempted to identify it^(^ 14 ^)^ or assess association with specific complications, such as pain^(^ 15 ^)^ and anemia^(^ 16 ^)^.

With regard to intercurrences during hemodialysis, investigations show, however, that they do not stop to explore their connection with HRQoL^(^ 17 ^-^ 18 ^)^. No specific studies on intercurrences were found after the end of the hemodialysis session and few were identified with general approaches on the symptoms^(^ 19 ^-^ 20 ^)^. Regarding adherence to drug therapy, there are few publications on the subject in this population that analyze the relationship with quality of life^(^ 21 ^)^.

In view of the aforementioned, there is a lack of knowledge regarding the association between HRQoL with complications of kidney disease, intercurrences during and after hemodialysis and adherence to drug therapy in chronic renal patients. Thus, this study aims to verify the association between the health-related quality of life of chronic renal patients on hemodialysis with sociodemographic, clinical, depression and medication adherence characteristics.

## Method

This is an exploratory, cross-sectional and analytical research, with a quantitative approach, developed from February to October 2017 in two renal units in Rio Grande do Sul, Brazil. One is a reference for the Northwest region, which integrates a philanthropic health care institution, and the other, a reference for the Missions region, a private administration clinic, for profit, but with greater demand from patients in the Unified Health System (Sistema Único de Saúde - SUS - in Portuguese).

The study population comprised 238 patients registered in the two units. Of these, 183 over 18 years old diagnosed with CKD and undergoing hemodialysis were included in the study; 20 were disregarded for not accepting to participate in the research. 18 patients did not meet the inclusion criteria - five minors, seven on peritoneal dialysis and six diagnosed with acute kidney disease. Six patients were also excluded because they had difficulty understanding the questions of the instruments. This difficulty was observed during the interview when they were unable to answer them clearly, even after the help of the interviewer or when they answered, several times, to something that was not related to what was being asked. Four were also excluded for having severe hearing or speech problems reported by the care team; four because they are occasionally undergoing hemodialysis in the services, because they are out on the town, traveling or another need and three due to the worsening of their health condition with displacement to another care unit.

Data collection, carried out by the researchers, took place with individual interviews during the hemodialysis sessions with the assistance of five nursing students and two pharmacy students trained for this purpose. Data was not collected from medical records. Sociodemographic and clinical questionnaires were used, as well as the Kidney Disease and Quality of Life Short-Form (KDQOL-SF^TM^), Back Depression Inventory (BDI) and Morisky Medication Adherence Scale - eight items.

The sociodemographic and clinical questionnaire comprised the following variables: age; gender; marital status; children; educational status; revenue; comorbidities; comorbidities, complications of kidney disease and intercurrences during and after hemodialysis. Among the comorbidities, the presence or absence of Systemic Arterial Hypertension (SAH) and Diabetes Mellitus (DM) for those being the main causes for the CKD in the world^(^ 3 ^)^.

The KDQOL-SF^TM^ is an instrument validated in Brazil that includes 80 items, the Short Form Health Survey (SF-36) and 43 more items on CKD. The SF-36 is divided into eight dimensions: physical functioning (ten items); limitations caused by physical health problems (four items); limitations caused by emotional health problems (three items); social functioning (two items); mental health (five items); pain (two items); vitality (four items); perceptions of general health (five items) and current health status compared to one year ago (one item). The items related to kidney disease are divided into 11 dimensions: symptoms/problems (12 items); effects of kidney disease on daily life (eight items); overload imposed by kidney disease (four items); working condition (two items); cognitive function (three items); quality of social interactions (three items); sexual function (two items); sleep (four items); social support scale (two items); dialysis team stimulation scale (two items) and patient satisfaction scale (one item). Another dimension comprises an item containing a scale from zero to ten for the assessment of health in general. The scores for each dimension range from zero to 100: being higher the score the better is the HRQoL^(^ 22 ^)^. The instrument also summarizes the score of the physical and mental component; the score of the first one derived from the items physical functioning, physical function, pain and general health and the second of the items vitality, social function, emotional role and mental health.

The BDI, validated in Portuguese and already used for the assessment of chronic kidney patients, consists of 21 items that include cognitive-affective, somatic aspects, guilt, life satisfaction, sleep disorders and health problems due to depression^(^ 23 ^)^. Each item is scored from zero to three: less than ten means no depression; ten to 18, indicative of mild depression; 19 to 29, moderate; 30 to 63, severe depression.

The Morisky Medication Adherence Scale - eight items was validated and adapted to Brazil and assesses the patient’s behavior regarding the usual drug use. It consists of seven questions with closed answers of a dichotomous yes/no character and the last question answered based on the options “never”, “almost never”, “sometimes”, “often” and “always”. Low adherence is considered to be those who answer affirmatively to more items from the test^(^ 24 ^)^.

The application of data collection instruments during the hemodialysis, although extensive, was not perceived by patients as uncomfortable, as the interview, as a form of interpersonal relationship, favored the perception that time in the dialysis unit passed more quickly.

For the operationalization of the analysis, the KDQOL-SF^TM^ data were inserted in a spreadsheet of the program Excel for Windows provided on-line by the RAND Health Care (https://www.rand.org/health-care/surveys_tools/kdqol.html), which automatically calculates scores by items and dimensions of the entire instrument. As this instrument does not have a cut-off point, the mean being displayed by the patients was used to be able to compare it with the results of other studies, which have also been using mean values.

The KDQOL-SF^TM^ together with sociodemographic, clinical data, BDI scores and the Morisky Medication Adherence Scale - eight items constituted a database in another Excel spreadsheet that, transposed to the Statistical Package for the Social Sciences (SPSS), version 21.0, were analyzed. Descriptive analysis was used with calculation of mean, standard deviation, median, minimum, maximum and interquartile range. Absolute and relative frequencies were also used. Joint frequency distributions were also performed and two study variables were simultaneously observed in order to identify a relationship between them using the chi-square test. A significance level of 0.05 was considered.

All ethical aspects that govern research with people have been respected. The research was approved by the Ethics Committee on December 16, 2016 under opinion No. 1,871,846 and CAAE: 62565316.6/0000.5322 were observed.

## Results

Of the 183 patients, 101 (55.2%) were 60 years of age or older, 116 (63.4%) were male, 119 (65.0%) lived with a partner, 159 (86.9%) , with children, 147 (80.3%) had low education and 166 (90.7%) were retired. Regarding the clinical aspects, in addition to the CKD, it was found that 68 (37.2%) had SAH and DM concomitantly; the others, one or another comorbidity and 111 (60.7%) had indications of depression, as can be checked in Table 1.

**Table 1:** Prevalence of indications of depression and comorbidities in chronic kidney patients during hemodialysis sessions (n = 183). Ijuí, RS, Brazil, 2017

Variables	Number of patients (%)
Indications of depression	111 (60.7)
Absence of depression	72 (39.3)
Mild depression	67 (36.6)
Moderate depression	41 (22.4)
Severe depression	3 (1.6)
Comorbidity	
Hypertension and Diabetes Mellitus	68 (37.2)
Hypertension	64 (35.0)
Diabetes Mellitus	21 (11.5)
Others	9 (4.9)
Absence	21 (11.5)

Remark: There was more than one response *per* participant

Table 2 shows the complications of patients related to CKD, intercurrences during and after hemodialysis and adherence to drug therapy. It is evident that, among complications, anemia was the most common in 127 patients (69.4%), followed by edema and cramps. During hemodialysis, hypotension and cramps were the most frequent intercurrences in 53.6% and 49.7% of patients, respectively. After the end of the session, 49.1% of the patients reported weakness; however, 28.4% mentioned the absence of symptoms.

**Table 2:** Complications of kidney disease previously reported by patients and intercurrences during and after hemodialysis sessions (n = 183). Ijuí, RS, Brazil, 2017

Complications and intercurrences	Number of patients (%)
Kidney disease complications	
Anemia	127 (69.4)
Edema	110 (60.1)
Cramp	96 (52.5)
Weakness	82 (44.8)
Pain	76 (41.5)
Cephalgia	59 (32.2)
Itching	43 (23.5)
Hypotension	38 (20.8)
Repetitive infections	28 (15.3)
Complications during hemodialysis	
Hypotension	98 (53.6)
Cramp	91 (49.7)
Pain	20 (10.9)
Intercurrences after hemodialysis	
Weakness	90 (49.1)
Absence of symptoms	52 (28.4)
Hypotension	43 (23.5)
Nauseas	41 (22.4)

Remark: There was more than one response per participant

Table 3 shows the mean scores of the HRQoL dimensions, minimum and maximum values. It appears that the worst scores are related to the dimensions Work status (19.40), Limitations due to physical problems (22.54) and Overload imposed by kidney disease (42.66). It is identified that the patients had a mean physical component (35.38) lower than the mental component (48.10). The general average of HRQoL was 62.61.

**Table 3:** Mean scores for the dimensions of quality of life related to the health of patients on hemodialysis. Ijuí, RS, Brazil, 2017

Dimensions	Mean	Standard Deviation	Median	Minimum	Maximum	Amplitude and Interquartile
Dialysis team support	88.93	19.53	100	0	100	100
Sexual function	84.24	24.41	100	0	100	100
Cognitive function	83.42	17.73	86.67	20	100	80
Quality of social interaction	81.75	18.26	86.67	20	100	80
Social support	80.69	26.27	83.33	0	100	100
Physical problems	75.35	16.48	79.17	16.67	100	83.33
Effects of the disease	70.98	20.11	75.00	9.38	100	90.62
Patient satisfaction	68.85	19.01	66.67	33.33	100	66.67
Sleep quality	68.12	23.05	72.50	0	100	100
Emotional wellbeing	68.09	24.74	76.00	4	100	96
Social role	67.08	28.69	75.00	0	100	100
Pain	64.81	30.70	67.5	0	100	100
Global health.	61.69	22.50	60.00	0	100	100
Vitality	57.38	23.24	60	0	100	100
General health	53.58	23.48	50.00	5	100	95
Limitations due to emotional problems	48.99	42.77	33.33	0	100	100
Functional capacity	43.74	29.53	40	0	100	100
Overload due to kidney disease	42.66	27.26	37.50	0	100	100
Limitations due to physical problems	22.54	33.77	0	0	100	100
Work situation	19.40	29.05	0	0	100	100
Summary of scores for the mental and physical component						
Mental component	48.10	11.35	48.8	19.12	67.48	48.36
Physical component	35.38	8.85	35.13	15.91	59.11	43.20

Figure 1 shows only sociodemographic variables, comorbidities and medication adherence significantly associated with scores below the general mean of HRQoL. It appears that sex and education are associated with the lowest means in a greater number of dimensions of HRQoL when compared with the other sociodemographic variables. Patients with symptoms of depression had lower means in all dimensions, except patient satisfaction.

**Figure 1:** Complications of kidney disease, intercurrences during and after hemodialysis and absence of symptoms significantly associated with values below the mean in the dimensions of quality of life presented by patients on hemodialysis. Ijuí, RS, Brazil, 2017

Variables	Below the mean quality of life dimensions
Characteristics of patients	
Age	Limitations due to emotional problems, Mental component
Marital Status	Work situation, Sexual function, Physical component
Gender	Limitations due to emotional problems, Cognitive function, Sexual function, Functional capacity, Mental component
Schooling	Social function, Cognitive function, Sexual function, Sleep quality
Income	Quality of social interaction, Quality of sleep
Comorbidities	
Depression	Effects of disease, Kidney disease burden, Quality of social interaction, Social support, Dialysis team support, Global health, Emotional well-being, Limitations due to emotional problems, Social function, Physical problems, Work situation, Cognitive function, Function sexual, Sleep quality, Functional capacity, Limitations due to physical problems, Pain, General health, Vitality, Mental component, Physical component
Hypertension	Work situation, Pain
Diabetes Mellitus	Sexual function, General health, Vitality, Functional capacity, Physical component
Drug adherence	Effects of the disease, Kidney disease overload, Quality of social interaction, Limitations due to emotional problems, Social function, Physical problems, Cognitive function, Limitations due to physical problems, General health, Vitality, Mental component

Remark: Association verified by chi-square test with p<0.05

Still in relation to the data contained in Figure 1, it appears that DM was correlated with more domains of HRQoL compared to SAH. In relation to medication adherence, an association was identified with half of the domains and mental component.

Sequentially, Figure 2 shows a significant association between complications of CKD, intercurrences during and after hemodialysis with scores below the mean in the dimensions of HRQoL. Among the complications, it is noted that repetitive infections negatively impact a greater number of dimensions (12) and mental component, followed by edema (eight dimensions and mental component).

**Figure 2:** Complications of kidney disease, intercurrences during and after hemodialysis and absence of symptoms significantly associated with values below the mean in the dimensions of quality of life presented by patients on hemodialysis. Ijuí, RS, Brazil, 2017

Variables	Below the mean quality of life dimensions
Kidney disease complications	
Cramp	Limitations due to emotional problems, Physical problems, Limitations due to physical problems
Cephalgia	Emotional well-being, Social function, Physical problems, Cognitive function, Functional capacity, Vitality, Mental component
Itching	Quality of social interaction, Physical problems
Hypotension	Effects of the disease, Physical problems, Work situation
Weakness	Quality of social interaction, Dialysis team support, Global health, Functional capacity, Limitations due to physical problems, Vitality
Pain	Effects of the disease, Physical problems, Work situation, Functional capacity, Pain, Vitality, Physical component
Repetitive infections	Effects of the disease, Kidney disease overload, Dialysis team support, Patient satisfaction, Emotional well-being, Limitations due to emotional problems, Social function, Physical problems, Sleep quality, Limitations due to physical problems, Pain, Health in general, Mental component
Anemia	Emotional well-being, Limitations due to emotional problems, Social function, Work situation, Limitations due to physical problems, Vitality, Mental component
Edema	Overload of kidney disease, Quality of social interaction, Emotional well-being, Social function, Work situation, Cognitive function, Sleep quality, Functional capacity, Mental component
Intercurrences during hemodialysis	
Cramp	Limitations due to physical problems, Pain, Health in general
Pain	Kidney disease overload, Physical problems, Functional capacity, Limitations due to physical problems, Vitality
Intercurrences after hemodialysis	
Weakness	Effects of disease, Kidney disease overload, Global health, Emotional well-being, Limitations due to emotional problems, Social function, Physical problems, Cognitive function, Functional capacity, Limitations due to physical problems, Pain, Vitality, Mental component, Physical component
Hypotension	Limitations due to physical problems
Nauseas	Effects of the disease, Overload of kidney disease, Limitations due to emotional problems, Physical problems, Sleep quality, Functional capacity, Pain, Vitality, Mental component
Absence of symptoms	Global health, Emotional well-being, Limitations due to emotional problems, Physical problems, Work situation, Functional capacity, Limitations due to physical problems, Vitality, Mental component, Physical component

Remark: Association verified by chi-square test with p<0.05

Still in Figure 2, among the intercurrences after hemodialysis, the weakness was related to the 12 dimensions of HRQoL and the physical component. The absence of symptoms was shown to be a protective factor and was associated with eight dimensions and physical and mental components.

## Discussion

Among the main findings of this study, the HRQoL perceived as regular by the patients stands out; depression, weakness after hemodialysis and complications related to CKD, particularly repetitive infections and edema, as factors that compromise a greater number of dimensions of quality of life and the important impact of medication adherence and the absence of symptoms in several domains. These results help to reduce the existing knowledge gap on the subject.

HRQoL shows low scores in the dimensions Work situation, Physical problems, Overload imposed by the disease, Functional capacity, Physical component and Mental component, higher value referring to the support of the dialysis team, similar to the results of a study in Portugal^(^ 25 ^)^. These data reveal that the well-being of patients on hemodialysis is compromised due to the physical, psycho-emotional state and the difficulties in maintaining a job and that support is an important tool to face this condition.

Regarding the indications of depression, more than half of the patients had symptoms, which is in line with the results of a study in India^(^ 26 ^)^. Depressive disorders in this population can be related to the worst clinical outcomes, comorbidities, complications of the disease and treatment, hospitalizations, increased length of hospital stay, abandonment of dialysis, mortality and reduced quality of life^(^ 9 ^,^ 27 ^)^.

In this investigation, indications of depression were associated with reduced scores in all dimensions of HRQoL, except patient’s satisfaction. A study in Egypt showed a similar result, except for the domain Limitations due to physical problems^(^ 28 ^)^. Despite the link between depression and unfavorable outcomes, a minority of the patients is adequately diagnosed and this situation may be due to the overlap of symptoms associated with uremia^(^ 7 ^)^. Measures aimed at reducing depressive symptoms become essential, including educational and problem-solving measures that can be developed by nursing professionals, including in dialysis units.

With regard to CKD complications, anemia, edema and cramps were reported by a greater percentage of patients; however, repetitive infections, edema, headache and anemia compromised a greater number of dimensions for the HRQoL. Similarly, research in Spain identified a negative relationship between somatic symptoms and quality of life, which are considered negative predictors of the physical and mental components^(^ 14 ^)^.

Infections increase the risk of hospitalization and are common in renal patients. A study in the United States found a crude infection incidence rate of 23.6 *per* thousand person/year, a higher number associated with lower glomerular filtration rates or a high urinary albumin/creatinine ratio, also related to the increased risk of mortality^(^ 29 ^)^. Both hospitalizations and infections decrease HRQoL and their management can be done with health-promoting, preventive and curative interventions.

As for edema, research in the United Kingdom has shown an association between chronic edema and quality of life, which is particularly reduced in terms of physical and emotional capacity and health in general^(^ 30 ^)^. It is considered that educational activities that favor self-care can positively impact the reduction of edema and improve HRQoL.

Pain is a common symptom among hemodialysis patients. In this research, a higher frequency of headache type was evidenced. In this sense, research in Western Pennsylvania found a painful sensation in 79% of patients, inversely linked to the Physical, mental components and global quality of life score^(^ 15 ^)^. During the dialysis session, the submitted data show that pain was related to physical, psycho-emotional and functional dimensions . A study in Greece showed cramping pain (61.2%) and headache (54.9%) during the session and point out that the subject’s self-efficacy in chronic pain conditions is impaired, with less efficient control of the painful sensation^(^ 31 ^)^.

Among the results, anemia also appeared as a common complication of CKD Iranian research found prevalence of anemia in 28.3% of the patients, 3% with hemoglobin levels below 8 g/dl^(^ 32 ^)^. Multicentric research, in Brazil, France, Japan and Germany, showed a worsening in the quality of life according to the severity of the anemia and indicated an association of this complication with the progression of kidney disease and mortality^(^ 16 ^)^. Data suggest that the adequate treatment of anemia, together with interventions that improve gastrointestinal symptoms and encourage compliance with the diet, and the provision of nursing care to prevent blood loss in hemodialysis may decrease the risk of this complication and its consequences.

Cramps, both as a complication of CKD and as a complication during hemodialysis, were reported as frequent by patients, with involvement of the lower limbs, hands and abdomen hypovolemia, hypomagnesemia, carnitine deficiency and elevated serum leptin levels appear to be involved in the event^(^ 17 ^)^. In this sense, research in Greece demonstrated an association between cramps and limitations due to physical problems, pain, general health and interpersonal relationships^(^ 31 ^)^. It is considered that the proper identification and management of this complication is a patient’s right and must integrate nursing care.

During the dialysis session, another prevalent complication was hypotension. Research in the Netherlands found a link between hypotension, increased cardiovascular morbidity and mortality and presence in more than half of dialysis sessions. The authors also revealed that patients with hypotension had lower pre-dialysis heart rates, low body weight and no residual renal function^(^ 18 ^)^.

After dialysis, the weakness was associated with the greater number of dimensions of HRQoL and may be linked to the waste of protein energy and low physical activity^(^ 33 ^)^. A study in England and Ireland evaluated the presence of symptoms and identified that just 3% of the patients did not have symptoms. There was a predominance of weakness in 78.0% of the patients, difficulty in movement in 66.0% and pain in 64%, all independently associated with a worse quality of life^(^ 19 ^)^. It is assessed that the weakness interferes with the recovery time after dialysis, the return to daily activities and, in this context, the recognition of the etiology by the health professional and the implementation of prevention and care actions can improve the patient’s conditions.

In relation to the absence of symptoms after dialysis, the results of this research showed it as a protective factor with influence in several domains of HRQoL, particularly in the Work Situation, Global Health, Functional Capacity, Limitation due to physical and emotional problems, Vitality, Well -Emotional being, Physical and mental components. This result reinforces the importance of Nursing and the multidisciplinary team to work to prevent, identify and reduce intercurrences^(^ 34 ^)^.

Adherence to drug therapy also had an impact on the HRQoL of patients evaluated in this research and was associated with the lowest mean scores related to the burden imposed by kidney disease, Effects of the disease, Quality of social interaction, Cognitive function, Health in general, Limitations due to emotional problems and physical, social function, vitality and mental component. Research in Iran has shown a significant positive relationship between treatment adherence and the overall quality of life score, as well as with all its dimensions. The authors emphasized the need for personalized educational interventions in order to sensitize the patient about his condition and the importance of adherence to treatment^(^ 35 ^)^. It is inferred that not using the medication properly increases the perception of the symptoms of the disease and the occurrence of damage to physical, psycho-emotional and social well-being.

As for the presence of comorbidities, a higher percentage of patients with arterial hypertension and diabetes was identified, concomitantly or with only one. The first was related to lower scores in the dimensions Work status and Pain; the second was associated with Vitality, Health in general, Functional capacity, Sexual function and Physical component. These diseases are considered the main causes of CKD^(^ 3 ^)^ and, regarding HRQoL, both negatively affect it^(^ 13 ^)^. It appears that having one or more diseases simultaneously increases the burden of physical, psycho-emotional symptoms and necessary care, which results in greater limitations, with a consequent worsening in the quality of life and evolution of the disease.

In the investigated patients, the sociodemographic profile is similar to that of research in São Paulo, except for age^(^ 36 ^)^. Regarding the association between HRQoL and sociodemographic characteristics, the age of less than 60 years had a lower score in the aspects Limitations due to emotional problems and Mental component, similar to an Australian study^(^ 10 ^)^. It is believed that the elderly have better adaptability or maturity to deal with the disease, respond better to stressors, which favors a more positive perception of HRQoL compared to younger people in the face of the CKD.

Regarding gender, women had lower scores on Cognitive Function, Sexual Function, Functional Capacity and Mental Component, similarly to the investigation in Turkey, which identified a reduced score in women regarding Symptoms, Effects of the disease and Physical and mental components^(^ 11 ^)^. The results of this research may be related to the lower percentage of muscle mass in women compared to men, which is linked to functional capacity. Likewise, there is evidence that decreased functional capacity has a worse cognitive performance^(^ 37 ^)^, which can be prevented or improved with physical activity. Impaired sexual function can be linked to aging, climacteric and the taboos that still exist regarding sex. Regarding the score of the mental component, it can be attributed to the reduction of estrogen levels in female aging that favor emotional changes and cultural values related to gender.

As for marital status, having a partner was associated with the worst scores in the dimension Work Situation, Physical Component and Sexual Function. A Brazilian study also showed less satisfaction in sexual function in individuals with a fixed partner^(^ 37 ^)^ in disagreement with another investigation, also Brazilian, which showed the partner as a synonym for greater social support^(^ 38 ^)^. It is inferred that patients with a partner feel supported to face the disease, however, they feel dependent on the partner in relation to the financial situation and would like to be in a better physical state to perform activities, including those related to the marital role.

Low schooling reflected in lower scores for cognitive function, quality of sleep, sexual function and social function. In this sense, a study in Brazil found that patients with a higher level of education (complete elementary school or more) had higher scores in several domains of HRQoL, while those with incomplete elementary school had a 4.3 times greater chance of impairment in social function^(^ 12 ^)^. Thus, it is understood that individuals with low education have little access to information and the ability to properly understand traumatic events, which can translate into greater concern, anxiety, insomnia and decrease their energy for other activities.

Income from retirement in CKD patients was linked to reduced quality of sleep and social interaction. On the other hand, research in Nepal showed that higher income was related to high scores in the Psychological, Environmental and Health domains in general^(^ 39 ^)^. It is suggested that people with less financial resources experience difficulties in coping with the costs of the disease, limiting their spending, including on leisure, which can interfere with sleep patterns and social interaction.

Assessing the HRQoL in patients with CKD undergoing hemodialysis is a complex task in view of the multiple factors involved in its perception and the difficulty to fully address it. However, the assessment of HRQoL favors the identification of the subjects’ needs for planning aimed at coping with the disease. In this sense, Nursing as a profession that requires direct contact with the patient must be able to identify factors that affect the quality of life of these patients, as well as to develop activities capable of reducing symptoms, improving physical and mental capacity, promoting self-care, that assist patients in adapting and coping with problems.

The results presented and discussed in this investigation contribute to the advancement of scientific knowledge by reducing gaps in the association between HRQoL with complications of kidney disease, intercurrences during and after hemodialysis and adherence to drug therapy in patients with CKD and by pointing out the potentially modifiable factors that reduce HRQoL and are able to be identified by professionals from the multiprofessional team, especially from Nursing, at the same time that they can become the focus of attention and care in order to promote health and well-being.

As a limitation of this research one highlights the methodology of the cross-sectional study, which allows only a specific perception of the patient’s conditions, although it is useful to identify needs and encourage the implementation of improvement interventions.

## Conclusion

The reduced HRQoL in chronic renal patients undergoing hemodialysis is associated, mainly, with depressive symptoms, complications of the disease such as repetitive infections, headache, pain and anemia, weakness after a dialysis session and low adherence to drug therapy. It was evident how much these factors interfere in the patients’ adaptation and that they can be modified, as long as the team responsible for care assumes this commitment. The effectiveness of this care can occur from the assessment of the conditions of the patients, planning and adequacy of dialysis, conventional, integrative or complementary interventions aimed at health, empowerment, self-care, physical improvement and psycho-emotional well-being. In relation to the public health, the appropriation of this knowledge can, positively, impact the development of policies and actions aimed at improving the quality of life of this population.
